# The Prevalence of Dental Anomalies in the Western Region of Saudi Arabia

**DOI:** 10.5402/2012/837270

**Published:** 2012-06-19

**Authors:** Ahmed R. Afify, Khalid H. Zawawi

**Affiliations:** Division of Orthodontics, Faculty of Dentistry, King Abdulaziz University, P.O. Box 80209, Jeddah 21589, Saudi Arabia

## Abstract

*Objective*. The aim of this cross-sectional study was to investigate the prevalence of dental anomalies that could be a cause of malocclusion in the western region of Saudi Arabia. *Materials and Methods*. A retrospective study of 878 digital orthopantomograms (OPGs) taken of patients, age ranging between 12 and 30 years, who presented to treatment at the Faculty of Dentistry, King Abdulaziz University, Jeddah, Saudi Arabia between 2002 and 2011. The OPGs and dental records were reviewed for congenitally missing teeth, supernumerary teeth, impactions, ectopic eruption, transposition, germination, fusion, dilacerations, taurodontism, dens in dent, and any other unusual conditions that can be assessed with OPG. *Results*. The prevalence of patient that exhibited at least one dental anomaly was 396 (45.1%) patients. The prevalence of congenitally missing teeth was 226 (25.7%), impacted teeth 186 (21.1%), dilacerated teeth 10 (1.1%), supernumerary teeth 3 (0.3%), odontoma 1 (0.1%), and taurodontism was also 1 case (0.1%) of the total radiographs reviewed. *Conclusions*. Congenitally missing teeth were found to be the most prevalent anomaly (25.7%), and the second frequent anomaly was impacted teeth (21.1%), whereas root dilacerations, supernumerary teeth, and taurodontism were the least frequent anomalies (1.1%, 0.3% and 0.1%, resp.).

## 1. Introduction

Dental anomalies in tooth number, shape, and position usually result in problems in maxillary and mandibular arch length and occlusion, which may greatly influence orthodontic treatment planning. The etiology of these conditions is usually attributed to certain genes in addition to some etiological events in the prenatal and postnatal periods that may result in anomalies in tooth size, shape, position, number, and structure [1–5].

Congenitally missing teeth constitute the most common developmental anomaly of the human dentition, occurring in approximately 25% of the population, and the wisdom tooth represents the most affected tooth (20.7%) [6]. Excluding third molars, the prevalence of tooth agenesis is approximately 4.3 to 7.8%, and the mandibular second premolars are the most commonly missing teeth, followed by the maxillary lateral incisors and maxillary second premolars [7]. Ethnic background was found to have an effect on the prevalence of tooth agenesis. Epidemiological studies revealed a lower prevalence of agenesis in the Black race compared to the White race, while Asians showed increased tooth agenesis compared to Whites [7]. Sexual differences were also reported in the prevalence of tooth agenesis, where women were more affected compared to men [7]. The unilateral occurrence is predominant, except in the case of maxillary lateral incisor agenesis, where bilateral agenesis is more common. The primary etiological factor of tooth agenesis is attributed to genetics [8] where the prevalence is higher in families of affected patients [9]. A mutation in the gene MSX1 of the chromosome 4 was identified in a family whose all members suffered from agenesis of second premolars and third molars [10]. Additionally, in a study that included monozygotic and dizygotic twins, a high frequency of agreement for tooth agenesis in monozygotic twins was found, while pairs of dizygotic twins showed disagreement for this dental anomaly [11]. 

Impacted teeth play a significant role in the etiology of different types of malocclusions. The permanent maxillary canines develop close to nasal cavity, far from the dental arch, and, therefore, have the longest eruption path compared to other permanent teeth. In about 1.5% of population, the canines show an ectopic eruption path towards the palate [6]. The orthodontic implication of this dental anomaly beside preventing the canines to erupt spontaneously, in a significant number of cases, could lead to root resorption of neighbouring teeth [12]. Peck et al. [13] postulated that genetics are the essential etiological factor in the palatally displaced canines. 

One or more dental anomalies can often be observed in the same patient. Studies on the patterns of association among seven types of dental anomalies in an untreated orthodontic population between the ages of 7 to 14 years found a significant reciprocal associations among five of the anomalies, a finding that suggests a common genetic origin. The prevalence of palatally displaced canine is greater in patients with microdontia. It was found that 34% of the patients with conical-shaped upper lateral incisors had palatally displaced canine [14]. When studying the association of maxillary first molars' agenesis with other dental anomalies in 32 Japanese orthodontic patients, the authors found that agenesis of maxillary first molars was associated with a higher prevalence of other permanent tooth agenesis [15]. In another study, the prevalence of agenesis of permanent teeth was 13-fold higher in subjects with absent third molars compared to subjects in whom third molars were present [16, 17].

Many researchers investigated the prevalence of various dental anomalies but relatively little have related it to orthodontic treatment. Therefore, the aim of this study was to investigate the prevalence of dental anomalies that could be a cause of malocclusion in the western Region of Saudi Arabia. 

## 2. Materials and Methods 

A total of 1255 records of patients who attended the Faculty of Dentistry, King Abdulaziz University between 2002 and 2011 were screened. Only 878 records satisfied the inclusion criteria. Only the records of Saudi patients who were between the ages of 12 to 30 years were selected. The exclusion criteria included patients who exhibited one or more of the following: any disease, trauma, or fracture of the jaws that may affect the normal growth of permanent dentition. Records of patients who presented with hereditary condition or syndromes that could cause dental anomaly, such as Down's syndrome or cleidocranial dysostosis were also rejected. Dental records and orthopantomograms (OPGs) were reviewed for the following dental anomalies: congenitally missing teeth (agenesis), supernumerary teeth, impaction, ectopic eruption, transposition, germination, fusion, dilacerations, taurodontism, dens in dente (Dens Evaginatus) and any other unusual condition. Digital orthopantomograms (OPGs) of these patients were examined in a standard manner under good lighting conditions, standardized screen brightness and resolution. The study was reviewed and approved by the Research Ethical Committee at the Faculty of Dentistry, King Abdulaziz University. 

Data was analyzed comparing males and females. The differences between the groups were tested using the Chi-square test. The level of significance was set at 5% (*P* < 0.05). The Statistical Package for Social Sciences (SPSS, version 11.01, Chicago, IL) was used.

## 3. Results

A total of 878 records met the inclusion criteria and were reviewed and examined. Of these 430 were males (49.0%) and 448 were females (51.0%). The prevalence of patients who had at least one dental anomaly was 45.1% of the total sample examined.  Table 1 shows the summary of the prevalence of dental anomalies found. A total of 226 (25.7%) patients had congenially missing teeth of which the third molar was missing in 185 patients (21.1%).

The prevalence of impaction was 21.2% (186 patients) and prevalence of third molar impaction was the highest (15.9%) compared to upper canine (3.3%), lower premolar (0.6%), and others (upper second premolar and lower second premolar, 1.4%). 

The prevalence of dilacerations was 1.1%, supernumerary teeth was 0.3%, taurodontism was 0.01% as well as odontoma.

When comparing all these dental anomalies between females and males, *𝒳* ^2^ showed that there were no significant differences between males and females with respect to the prevalence of all dental anomalies, *P* > 0.05.

## 4. Discussion

Orthodontic patients have been reported to have high rates of dental anomalies [9, 12–14]. Inadequate consideration of these dental anomalies can complicate orthodontic treatment; therefore, their presence should be thoroughly investigated during orthodontic diagnosis and carefully considered during treatment planning. 

This study was done to detect the prevalence of dental anomalies in the western region of Saudi Arabia that could be a cause of malocclusion. One can argue that the selected sample could only represent a limited population. The Faculty of Dentistry receives patients not only for the city of Jeddah but also from the surrounding suburban areas as well as neighboring cities. Therefore, it is fair to state that the selected sample could represent the geographic western region of Saudi Arabia. Dental anomalies may be expressed with different degrees of severity from the mildest developmental delay to the most severe tooth agenesis manifestation; dental anomalies may be expressed as microdontia, changes in dental morphology and ectopias [3]. Several studies have suggested a genetic and hereditary background in the etiology of dental anomalies of number, size, position, as well as timing of development. This was derived from studies in families, monozygotic twins, and from the frequent observation of associations of certain dental anomalies [9–11, 14]. 

The prevalence of dental anomalies reported in this study was rather high, 45.1%. This could be attributed to a large extent to the anomalies of the wisdom teeth. A high prevalence of congenitally missing and impaction of wisdom teeth is reported in this study. One limitation to this finding is that the impactions were not classified (e.g., partial or fully impacted) and the angulation of impaction was not taken into consideration. 

The association between the unilateral agenesis of the maxillary lateral incisor and the microdontia of the contralateral one, often observed clinically, well explains this condition. In this case, the same genetic defect, which determined the agenesis, has an incomplete expression in the opposite side of the dental arch, causing microdontia. However, the associations between the dental anomalies are not restricted to this usual example. There are many more interactions between different dental anomalies. The clinical implications are important because the early diagnosis of a given dental anomaly can alert the professional to the possible development of other associated dental anomalies in the same patient or family, permitting early diagnosis and timely orthodontic management.

The data in this study indicate the prevalence of tooth impaction is similar to the data reported in earlier studies while other studies report different rates. The current data showed that the incidence of impaction to be 21.2%, and this is somewhat higher than what was reported previously [18–22]. In this study, maxillary canines were the most commonly impacted teeth, excluding third molars. Furthermore, the prevalence of impacted cuspid was 3.3%, and this figure is similar to findings of Aydin et al. [23] and Zahrani [24] who reported 3.6% and 3.6% cuspid impaction, respectively. However, Fardi et al. [25] reported significantly lower figures in which he only observed 8.8% of cases exhibiting cuspid impaction. 

Previous reports demonstrated the rarity of impacted cuspids in the mandible and this is also confirmed in the present study [19, 26–28]. In fact, none of the cases reviewed showed mandibular cuspid impacted. Grover and Lorton [19] examined 5,000 panoramic radiographs and found 142 cases having impacted cuspids in the maxilla (2.8%) and only 11 in the mandible, 0.2%. In another study of 1,000 Turkish patients, the incidence of maxillary cuspid impaction was 2.9%, while the incidence of impacted mandibular cuspid was 0.3% [29]. Shah et al. [20] observed only eight impacted mandibular cuspids among 7,886 patients (0.1%). Early detection of impacted teeth is of paramount importance from a therapeutic point of view. Impacted teeth result in many complications, and their early detection is imperative. 

Previous reports showed that the range of the prevalence of supernumerary teeth was 0.1–3.8% of the population [30–32]. The current finding, however, was 0.3%, a finding that is not in concert with what was reported by Fardi et al. [25] in which the prevalence of supernumerary teeth was 1.8%. 

Several studies reported the incidence of the supernumerary teeth in different populations. Bäckman and Wahlin [30] conducted a clinical study by examining 739 Caucasian children and found 14 cases (1.9%) of at least one supernumerary tooth, and the majority of the supernumerary teeth reported were mesiodens. Salem [32] investigated the incidence of supernumerary teeth in a sample of 2,393 Saudi Arabian children and found that only 0.5% sample studied had at least one supernumerary tooth. 

Several investigators indicated a higher prevalence of supernumerary teeth in males compared to females. In this study the male-to-female ratio was 1 : 2, which is in accord with the ratio reported for the Caucasians [31] but in disagreement with the ratios reported in other studies [25, 31, 33]. This could be due to the small number of observed supernumerary cases (i.e., 2 females and one male). 

## 5. Conclusions

- Congenitally missing teeth were found to be the most prevalent anomaly (25.7%), and the second frequent anomaly was impacted teeth (21.1%).
- Root dilacerations, supernumerary teeth, and taurodontism were the least frequent anomalies observed (1.1%, 0.3%, and 0.1% resp.).
- There were no significant differences in the prevalence of different dental anomalies between males and females.

## Figures and Tables

**Table 1:** Summary of prevalence of patients with different dental anomalies according to gender (number and percentage).

	Males (*n* = 430)	Females (*n* = 448)	Both (*n* = 878)
	No. (%)	No. (%)	No. (%)
Congenitally missing teeth	107 (24.9)	119 (26.6%)	226 (25.7%)
(i) Third molars	86 (20.0)	99 (22.1%)	185 (21.1%)
(ii) Lower premolars	7 (1.6)	6 (1.3)	13 (1.5)
(iii) Upper canines	3 (0.7)	2 (0.4)	5 (0.6)
(iv) Other	11 (2.6)	12 (2.7)	23 (2.6)

Impactions	10 (23.3)	86 (19.2)	186 (21.2)
(i) Third molars	74 (17.2)	66 (14.7)	140 (15.9)
(ii) Upper canines	16 (3.7)	13 (2.9)	29 (3.3)
(iii) Lower premolars	1 (0.2)	4 (0.9)	5 (0.6)
(iv) Others	9 (2.1)	3 (0.7)	12 (1.4)

Ectopic eruption	1 (0.5)	1 (0.2)	2 (0.3)
(i) Third molars	1 (0.2)	1 (0.2)	2 (0.2)
(ii) Lower premolars	1 (0.2)	0 (0.0)	1 (0.1)

Other anomalies	6 (1.4)	6 (1.3)	12 (1.4)
(i) Dilaceration	6 (1.4)	4 (0.9)	10 (1.1)
(ii) Supernumerary	2 (0.5)	1 (0.2)	3 (0.3)
(iii) Odontoma	0 (0.0)	1 (0.2)	1 (0.1)
(iv) Taurodontism	0 (0.0)	1 (0.2)	1 (0.1)

## References

[1] Basdra EK, Kiokpasoglou M, Stellzig A (2000). The Class II division 2 craniofacial type is associated with numerous congenital tooth anomalies. *European Journal of Orthodontics*.

[2] Baydaş B, Oktay H, Dağsuyu IM (2005). The effect of heritability on Bolton tooth-size discrepancy. *European Journal of Orthodontics*.

[3] Garn SM, Lewis AB, Kerewsky RS (1965). X-linked inheritance of tooth size. *Journal of Dental Research*.

[4] Kotsomitis N, Dunne MP, Freer TJ (1996). A genetic aetiology for some common dental anomalies: a pilot twin study. *Australian Orthodontic Journal*.

[5] Sofaer JA (1979). Human tooth-size asymmetry in cleft lip with or without cleft palate. *Archives of Oral Biology*.

[6] Garib DG, Peck S, Gomes SC (2009). Increased occurrence of dental anomalies associated with second-premolar agenesis. *Angle Orthodontist*.

[7] Polder BJ, Van’t Hof MA, Van Der Linden FPGM, Kuijpers-Jagtman AM (2004). A meta-analysis of the prevalence of dental agenesis of permanent teeth. *Community Dentistry and Oral Epidemiology*.

[8] Shapira Y, Finkelstein T, Shpack N, Lai YH, Kuftinec MM, Vardimon A (2011). Mandibular second molar impaction. Part I: genetic traits and characteristics. *American Journal of Orthodontics and Dentofacial Orthopedics*.

[9] Mossey PA (1999). The heritability of malocclusion: part 2. The influence of genetics in malocclusion. *British Journal of Orthodontics*.

[10] Vastardis H (2000). The genetics of human tooth agenesis: new discoveries for understanding dental anomalies. *American Journal of Orthodontics and Dentofacial Orthopedics*.

[11] Markovic M (1982). Hypodontia in twins. *Swedish Dental Journal*.

[12] Ericson S, Kurol J (2000). Resorption of incisors after ectopic eruption of maxillary canines: a CT study. *Angle Orthodontist*.

[13] Peck S, Peck L, Kataja M (1994). The palatally displaced canine as a dental anomaly of genetic origin. *Angle Orthodontist*.

[14] Baccetti T (1998). A controlled study of associated dental anomalies. *Angle Orthodontist*.

[15] Abe R, Endo T, Shimooka S (2010). Maxillary first molar agenesis and other dental anomalies. *Angle Orthodontist*.

[16] Garn S, Lewis AB (1962). The relationship between third molar agenesis and reduction in tooth number. *The Angle Orthodontist*.

[17] GARN SM, LEWIS AB, VICINUS JH (1962). Third molar agenesis and reduction in the number of other teeth. *Journal of Dental Research*.

[18] Bedoya MM, Park JH (2009). A review of the diagnosis and management of impacted maxillary canines. *Journal of the American Dental Association*.

[19] Grover PS, Lorton L (1985). The incidence of unerupted permanent teeth and related clinical cases. *Oral Surgery, Oral Medicine, Oral Pathology*.

[20] Shah RM, Boyd MA, Vakil TF (1978). Studies of permanent tooth anomalies in 7,886 Canadian individuals. I: impacted teeth. *Dental Journal*.

[21] Thilander B, Myrberg N (1973). The prevalence of malocclusion in Swedish schoolchildren. *Scandinavian Journal of Dental Research*.

[22] Gunduz K, Acikgoz A, Egrioglu E (2011). Radiologic investigation of prevalence, associated pathologies and dental anomalies of non-third molar impacted teeth in Turkish oral patients. *The Chinese Journal of Dental Research*.

[23] Aydin U, Yilmaz HH, Yildirim D (2004). Incidence of canine impaction and transmigration in a patient population. *Dentomaxillofacial Radiology*.

[24] Zahrani AA (1993). Impacted cuspids in a Saudi population: prevalence, etiology and complications. *Egyptian Dental Journal*.

[25] Fardi A, Kondylidou-Sidira A, Bachour Z, Parisis N, Tsirlis A (2011). Incidence of impacted and supernumerary teeth-a radiographic study in a North Greek population. *Medicina Oral, Patologia Oral y Cirugia Bucal*.

[26] Celikoglu M, Kamak H, Oktay H (2010). Investigation of transmigrated and impacted maxillary and mandibular canine teeth in an orthodontic patient population. *Journal of Oral and Maxillofacial Surgery*.

[27] Yavuz MS, Aras MH, Büyükkurt MC, Tozoglu S (2007). Impacted mandibular canines. *Journal of Contemporary Dental Practice*.

[28] Kramer RM, Williams AC (1970). The incidence of impacted teeth. A survey at Harlem Hospital. *Oral Surgery, Oral Medicine, Oral Pathology*.

[29] Sağlam AA, Tüzüm MSMS (2003). Clinical and radiologic investigation of the incidence, complications, and suitable removal times for fully impacted teeth in the Turkish population. *Quintessence International*.

[30] Bäckman B, Wahlin YB (2001). Variations in number and morphology of permanent teeth in 7-year-old Swedish children. *International Journal of Paediatric Dentistry*.

[31] Luten JR (1967). The prevalence of supernumerary teeth in primary and mixed dentitions. *Journal of Dentistry for Children*.

[32] Salem G (1989). Prevalence of selected dental anomalies in Saudi children from Gizan region. *Community Dentistry and Oral Epidemiology*.

[33] Ferrés-Padró E, Prats-Armengol J, Ferrés-Amat E (2009). A descriptive study of 113 unerupted supernumerary teeth in 79 pediatric patients in Barcelona. *Medicina Oral, Patologia Oral y Cirugia Bucal*.

